# Does education offset the effect of maternal disadvantage on childhood anaemia in Tanzania? Evidence from a nationally representative cross-sectional study

**DOI:** 10.1186/s12887-019-1465-z

**Published:** 2019-04-03

**Authors:** Olaide O. Ojoniyi, Clifford O. Odimegwu, Emmanuel O. Olamijuwon, Joshua O. Akinyemi

**Affiliations:** 1 Implementation Science Department, The Wits Reproductive Health & HIV Institute, P.O Box 2193, Johannesburg, South Africa; 2 Demography and Population Studies Programme, Schools of Social Sciences and Public Health, University of the Witwatersrand, Johannesburg, South Africa; 3 Department of Statistics and Demography, Faculty of Social Sciences, University of Eswatini, Kwaluseni, Eswatini Swaziland; 4 Department of Epidemiology and Medical Statistics, College of Medicine, University of Ibadan, Ibadan, Nigeria

**Keywords:** Anaemia, Under-five children, Maternal characteristics, *TDHS-MIS*, Tanzania

## Abstract

**Background:**

Despite being preventable, anaemia is a major public health problem that affects a sizable number of children under-five years globally and in Tanzania. This study examined the maternal factors associated with the risk of anaemia among under-five children in Tanzania. We also assessed whether higher maternal education could reduce the risks of anaemia among children of women with poor socio-economic status.

**Methods:**

Data was drawn from the 2015–16 Tanzania demographic and health survey and malaria indicator survey for 7916 children under five years. Adjusted odds ratios were estimated by fitting a proportional odds model to examine the maternal risk factors of anaemia. Stratified analysis was done to examine how the relationship differed across maternal educational levels.

**Results:**

The findings revealed that maternal disadvantage evident in young motherhood [AOR:1.43, 95%CI:1.16–1.75], no formal education [AOR:1.53, 95%CI:1.25–1.89], unemployment [AOR:1.31, 95%CI:1.15–1.49], poorest household wealth [AOR:1.50, 95%CI:1.17–1.91], and non-access to health insurance [AOR:1.26, 95%CI: 1.03–1.53] were risk factors of anaemia among children in the sample. Sub-group analysis by maternal education showed that the risks were not evident when the mother has secondary or higher education. However, having an unmarried mother was associated with about four-times higher risk of anaemia if the mother is uneducated [AOR:4.04, 95%CI:1.98–8.24] compared with if the mother is currently in union.

**Conclusion:**

Findings from this study show that a secondary or higher maternal education may help reduce the socio-economic risk factors of anaemia among children under-5 years in Tanzania.

## Background

Anaemia, particularly among children under 5 years, is a public health problem of serious concern. In East Africa, approximately 75% of under-five children suffer from anaemia [1]. In regions within Tanzania, the prevalence of anaemia among under-five children ranges between 44 and 76% [2]. In 2010, the Tanzania demographic and health survey reported the prevalence of anaemia in the Lake Zone to be 55%. In most health facilities in Tanzania, severe anaemia is among the causes of admission and mortality in the paediatrics’ ward [3].

Poor nutritional status, micro-nutrient deficiencies, intestinal worms, HIV infection, haematological malignancies and chronic diseases such as sickle cell disease are known to be contributing factors of the high prevalence of anaemia [1]. Its implications for health, as well as social and economic development, are diverse. Among children, it weakens their mental and physical development resulting in poor academic performance and employability in later years [3].

Over the years, diverse intervention programs such as food fortification, vitamin A for under-five children and iron folate supplement for pregnant women have been implemented with the aim of reducing childhood anaemia in Tanzania [3]. Despite these interventions, the prevalence of anaemia remains high. Given its prevalence and the developmental challenges that it poses for children, understanding the risk factors of anaemia is expected to drive interventions and inform existing prevention measures towards achieving the Post 2015 Sustainable development goal 3 for improvement in health and well-being.

Several studies have examined the socio-demographic factors associated with child health and survival using malnutrition and mortality as key proxies for child health. In the absence of urgent treatment, anaemic children also suffer severe complications [4]. Although some factors such as malaria, HIV infections and malnutrition are known to increase anaemia risk among children, other potential risk factors such as maternal education and other socio-economic characteristics of the mother may increase the risk of anaemia among children [5–7].

Evidence of the importance of maternal education continues to emerge [6, 8–10]. Studies have shown that maternal education can reduce risks of poor child health through higher health knowledge, adherence to recommended feeding practices for children, and increased command over resources [10, 11]. Highly educated mothers can better understand printed and audio health information, convey their children’s health needs at health facilities and better understand complex treatment regimens [11]. Given these benefits, it is likely that disadvantages in other socio-economic characteristics may have little effects on the children of better-educated mothers.

Yet, in Tanzania and in part of sub-Saharan Africa where anaemia is prevalent, evidence of the benefit of maternal education in reducing the risks associated with maternal disadvantage are scarce. As a result, we examined if maternal education might reduce the risk of anaemia in under-five children whose mothers are disadvantaged in other socio-economic indices. To clearly understand this relationship, we analyzed the recent dataset from the Tanzania demographic and health survey and malaria indicator survey to illuminate the prevalence and associated factors of anaemia across selected socio-demographics. Our study builds upon Mosley and Chen’s 1984 analytical framework for the study of maternal and child survival in developing countries [6]. We argue that the risk of anaemia in children may be reduced if the socio-demographic and economic characteristics relating to the mothers are improved. An awareness of this relationship is expected to facilitate improved targeted interventions for reducing the risks of anaemia among under-five children in Tanzania.

## Data and methods

Data for this study was drawn from the children recode dataset of the 2015–16 Tanzania demographic, health and malaria indicator survey. The dataset contains information that may be used to monitor and evaluate the demographic and health indicators of children under 5 years.

In order to provide estimates intended to be representative of the entire country, a two-stage sample design was used. This sampling design allowed the estimation of indicators for each of the 30 regions of the country. The first stage of the sampling design, involved the selection of 608 clusters, consisting of enumeration areas delineated for the 2012 Tanzania Population and Housing Census [12]. In the second stage, 22 households from each of the clusters was systematic selected. This was done after a complete households listing was carried out for all 608 selected clusters in the country [12]. This yielded a total representative probability sample of 10,233 children under 5 years born to 13,266 women who completed the interviews and reside in any of the 13,376 households selected for participation in the survey. In all the households, with the parent’s or guardian’s consent, children age 6–59 months were tested for anaemia and malaria.

A sub-sample of 2219 children who did not participate in the anemia module were further excluded from analysis. This comprised of children who were not alive or physically present at the time of data collection, children whose parents or guardian refused, and those who were less than 6 months of age at the time of data collection. We also excluded 98 children with missing information on key demographic, maternal, and household characteristics. This led to a final analytic sample of 7916 children under five (6–59 months) years in Tanzania who had complete information on all socio-demographic variables and whose anthropometric data and anaemia data were collected.

### Variable description

#### The outcome variable

The main outcome variable for this study was anaemia status, adjusted for altitude measured in grams per deciliter (g/dl). The variable was categorized by haemoglobin levels ranging from no anaemia (0), mild (1) moderate (2), and severe anaemia (3). During the survey, all children age 6–59 months living in the selected households were assessed for anaemia through finger prick or, in the case of young children, heel prick blood testing using the HemoCue blood haemoglobin testing system which measures the concentration of haemoglobin in the blood. The anaemia cutoff points used in this study were those recommended by the World Health Organization (WHO) for children [13]. Haemoglobin levels below 7.0 g/dl were considered as severe anaemia, levels between 7.1 g/dl and 9.9 g/dl were considered as moderate anaemia and levels between 10.0 g/dl and 10.9 g/dl are considered as mild anaemia for all children in the sample [14, 15]. The mothers of children whose anaemia level was severe were asked whether information on their child’s health can be given to a doctor at a specified health facility for follow up.

#### Predictor variables

The predictor variables included in the study are socio-economic and demographic variables namely: mother’s educational attainment *(no education, primary, secondary+)*; age group in years *(15–24, 25–34, 35–49)*; marital status *(not married, currently married and formerly married)*; employment status *(not working and currently working)*; place of residence *(mainland-urban, mainland-rural, Zanzibar-urban, and Zanzibar-rural)*; mother’s body mass index *(underweight, normal, overweight, and severe obesity).* Household wealth status was assessed using a principal component analysis that combined scores for each household based on the number and kinds of consumer goods they own, ranging from a television to a bicycle or car, plus housing characteristics, such as source of drinking water, toilet facilities, and flooring materials. Households were subsequently ranked and divided into quintiles of five equal categories *(poorest, poorer, middle, richer and richest)* with each accounting for 20% of the population [12]. Health insurance *(no health insurance and has health insurance)*. Child’s characteristics included child sex *(male and female)*; child age in months *(6–23, 24–47, and 48–59 months)*, birth type *(single and multiple)*, number of siblings and Child’s BMI *(low, normal, overweight, obese)*.

### Statistical analysis

Frequency distributions were used to describe the profile of children in the sample. The outcome variable was also tabulated against the predictor variables and covariates to assess the prevalence of anaemia in the sample. Chi-square (χ^2^) tests were used to determine statistically significant differences in the prevalence of anaemia across the predictor variables. We estimated adjusted odds ratios (AORs) and 95% confidence intervals (CIs) for the association between the predictors and the outcome using a proportional odds model, a regression model for an ordinal outcome variable [16]. This model uses cumulative probabilities to a threshold, thereby making the whole range of ordinal categories binary at that threshold. All model diagnostics inclusive of Wald, Brant, and Score test provided evidence that the model fits reasonably for our data. Robust standard errors were estimated to account for sampling errors.

Furthermore, we considered how the relationship between the predictors and the risk of anaemia among children may differ by maternal educational attainment by fitting a proportional odds regression model stratified by maternal level of education. G-Power version 3.1.9.2 was used for Post-hoc power estimation to ascertain that there is sufficient sample size for stratified analysis and the result showed that the statistical power was greater than 80% [17]. Interpretation of the results was done using odds ratios (OR) with a confidence interval of 95%. Results for analysis were weighted to adjust for sampling error and the clustering of the sample. Data management and analyses were performed in Stata/MP version 15.1 (StataCorp, College Station, USA).

## Results

### Descriptive profile of study sample

The total sample for this study comprised of 7916 children under 5 years in Tanzania. As presented in Table 1, slightly above one-quarter (28%) of the children were born to adolescent and young women between 15 and 24 years of age. Only about 5% of the children were born to unmarried women while the majority (85%) were born to women who are currently married. More than half of the children have a mother with primary education while almost one-quarter have a mother with no formal education. Most (80%) of the children have a mother who is currently working. About 25% of the children reside in the urban area while the rest reside in the rural areas of the Mainland (73%) and the Zanzibar (2.0%). Less than one-tenth of the children had medical insurance. About 68% of the children had a mother with a normal BMI between (18.5–24.9) while about 25% had a mother who is either overweight or obese. Overall, 46% of the children reside in the poorest or poorer household and about 34% reside in the richer or richest household. The majority of the children (97%) were single births and about 61% of the children were 2 years or older. Only about three-quarter of the children have a normal BMI according to the WHO growth standard.

**Table 1:** Descriptive Characteristics of the Study Population *(Source: TDHS, 2015-16)*

Characteristics	Sample *n* = 7916	Percentage %
Mother’s Age Group		
15–24	2153	28.2
25–34	3567	45.2
35–49	2196	26.6
Marital Status		
Not Married	346	4.9
Currently Married	6768	84.5
Formerly Married	802	10.6
Educational Attainment		
No Education	1742	21.7
Primary	4758	64.5
Secondary+	1416	13.8
Employment Status		
Not Working	1655	20.0
Currently Working	6261	80.0
Number of Siblings	*median* 3	*S.Dev* 2.54
Place of Residence		
Mainland - Urban	1550	24.8
Mainland - Rural	5193	72.6
Zanzibar - Urban	219	0.7
Zanzibar - Rural	954	1.9
Health Insurance		
No Health Insurance	7358	92.4
Health Insurance	558	7.6
Mother’s BMI		
Under Weight	560	6.8
Normal	5300	68.1
Overweight	1864	22.6
Severe Obesity	192	2.4
Wealth Status		
Poorest	1810	24.4
Poorer	1658	22.0
Middle	1565	19.6
Richer	1628	18.2
Richest	1255	15.8
Birth Type		
Single	7673	96.9
Multiple	243	3.1
Child’s Sex		
Male	3971	50.6
Female	3945	49.4
Child’s Age		
6–23 months	3052	38.8
24–47 months	3297	41.8
48–59 months	1567	19.4
Child’s BMI		
Low	989	3.7
Normal	6092	76.0
Overweight	1185	15.7
Obese	339	4.6

Frequency distributions are unweighted while percentages are weighted

### Prevalence of Anaemia across maternal socio-demographic characteristics

In Table 2, we examined the prevalence of anaemia across selected characteristics. More than half of the children aged 6–59 months in the sample were anaemic with about 2% manifesting severe anaemia. The prevalence of anaemia is significantly different (*p* < 0.05) across maternal characteristics, excluding maternal marital status. We found that anaemia was more common among children of adolescent and young women (64%) and lowest among children of middle-aged (35–44 years) women (54%). Both severe (3%) and mild/moderate (64%) anaemia are more common among children of women with no formal education although more than half (55%) of the children of women with secondary or higher education are anaemic. Severe anaemia is more common among children whose mothers resides in the mainland while mild/moderate anaemia is more common among children whose mothers reside in the Zanzibar. More than half (60%) of the children whose mothers do not have a health insurance are anaemic.

**Table 2:** Prevalence of Anaemia and Associated Factors among Children Under-Five Years *(TDHS-MIS, 2015-16)*

Socio-Demographic Characteristics	% with any Anaemia	Anaemia severity		
		% with mild /moderate Anaemia	% with Severe Anaemia	*p-value*
Mother’s Age Group				
15–24	64.0	61.9	2.2	0.000
25–34	57.7	56.1	1.7	
35–49	54.4	53.1	1.3	
Marital Status				
Not Married	64.7	63.7	1.0	0.182
Currently Married	58.2	56.4	1.8	
Formerly Married	59.5	57.7	1.7	
Educational Attainment				
No Education	66.4	63.8	2.7	0.000
Primary	56.7	55.2	1.5	
Secondary+	55.2	54.1	1.1	
Employment Status				
Not Working	63.5	61.1	2.4	0.001
Currently Working	57.4	55.9	1.6	
Number of Siblings	3	3	3	0.581
Place of Residence				0.000
Mainland - Urban	54.4	53.4	1.1	0.000
Mainland - Rural	59.8	57.8	2.0	
Zanzibar - Urban	63.9	63.6	0.3	
Zanzibar - Rural	67.0	66.1	0.9	
Wealth Status				
Poorest	63.8	61.4	2.3	0.000
Poorer	61.2	58.5	2.7	
Middle	59.8	59.0	0.8	
Richer	53.4	52.1	1.3	
Richest	51.6	50.6	1.0	
Health Insurance				
No Health Insurance	59.5	57.7	1.8	0.000
Health Insurance	48.0	47.4	0.6	
Mother’s BMI				
Under Weight	61.2	58.1	3.1	0.000
Normal	61.4	59.5	1.9	
Overweight	51.2	50.2	0.9	
Severe Obesity	43.2	42.2	0.9	
Birth Type				
Single	58.6	56.9	1.7	0.181
Multiple	60.8	57.2	3.6	
Child’s Sex				
Male	60.2	58.4	1.9	0.025
Female	57.0	55.4	1.5	
Child’s Age				
6–23 months	75.3	72.5	2.8	0.000
24–47 months	50.8	49.7	1.1	
48–59 months	42.3	41.5	0.8	
Child’s BMI				
Low	68.4	64.4	4.0	0.000
Normal	57.2	55.5	1.7	
Overweight	62.1	60.7	1.5	
Obese	62.4	61.0	1.3	
Sample	4612 (58.6%)	4493 (56.9%)	119 (1.7%)	

The prevalence of anaemia is also higher among children of underweight mothers (61%) and lowest among children whose mothers are obese (43%). Across wealth status, almost two-thirds of the children whose mother reside in the poorest households are anaemic with almost 2% manifesting severe anaemia. Slightly more than half of those whose mothers resides in the richest households are also anaemic.

Examining the prevalence of anaemia across selected child characteristics, we found a statistically significant difference in the prevalence of anaemia by child’s sex (*p* < 0.05), child’s age (*p* < 0.05), and child’s body mass index (*p* < 0.05). Anaemia was more prevalent among children with multiple births (61%), boys (60%), children under 2 years (75%) and children with a low body mass index (68%).

### Maternal socio-demographic factors associated with the risk of Anaemia

Results from Table 3 show the socio-demographic characteristics associated with the risk of anaemia among children under 5 years while adjusting for covariates. The combined risk of severe, mild or moderate anaemia is higher among children of adolescent and young women [AOR: 1.43, 95%CI: 1.16–1.75] as well as those of women aged 25–34 years [AOR: 1.22, 95%CI: 1.05–1.42] compared to children of women who are 35 years or older. Maternal educational attainment is also significantly associated with the risk of anaemia. Children of women with no formal education [AOR: 1.53, 95%CI: 1.25–1.89] are significantly more likely to be anaemic compared to the children of women with secondary or higher education. Children whose mothers are not working [AOR: 1.31, 95%CI: 1.15–1.49] are also at a higher risk of anaemia compared to children whose mother are currently working. A higher number of siblings is associated with a higher risk of anaemia among the children [AOR: 1.05, 95%CI: 1.01–1.08].

**Table 3:** Risk Factors of Anaemia Among Children Under-Five Years in Tanzania Stratified by Maternal Educational Attainment *(TDHS-MIS, 2015–16)*

Socio-Demographic Characteristics	All Children Sample (*n* = 7916)	No Education (*n* = 1742)	Primary (*n* = 4758)	Secondary+ (*n* = 1416)
	Adjusted Odd Ratios [95% CI]			
Mother’s Age Group				
15–24	1.43*** [1.16,1.75]	1.15 [0.74,1.79]	1.55*** [1.20,2.00]	1.62 [0.90,2.89]
25–34	1.22* [1.05,1.42]	1.29 [0.95,1.76]	1.21* [1.00,1.46]	1.55 [0.97,2.48]
35–49	Reference	Reference	Reference	Reference
Marital Status				
Not Married	1.09 [0.87,1.38]	4.04*** [1.98,8.24]	1.08 [0.80,1.44]	0.86 [0.57,1.31]
Currently Married	Reference	Reference	Reference	Reference
Formerly Married	1.04 [0.89,1.22]	0.95 [0.68,1.32]	1.07 [0.87,1.31]	1.13 [0.71,1.81]
Educational Attainment				
No Education	1.53*** [1.25,1.89]			
Primary	1.06 [0.90,1.26]			
Secondary+	Reference			
Employment Status				
Not Working	1.31*** [1.15,1.49]	1.62** [1.21,2.16]	1.21* [1.03,1.43]	1.34 [0.97,1.85]
Currently Working	Reference	Reference	Reference	Reference
Number of Siblings	1.05** [1.01,1.08]	1.05 [0.98,1.12]	1.06** [1.02,1.10]	0.97 [0.86,1.10]
Place of Residence				
Mainland - Urban	Reference	Reference	Reference	Reference
Mainland - Rural	0.89 [0.76,1.05]	0.65* [0.44,0.97]	0.95 [0.78,1.17]	0.99 [0.66,1.50]
Zanzibar - Urban	1.76*** [1.30,2.38]	0.49 [0.22,1.08]	1.87 [0.98,3.56]	2.28*** [1.48,3.54]
Zanzibar - Rural	1.39*** [1.16,1.66]	0.9 [0.58,1.38]	1.38* [1.05,1.82]	1.84*** [1.30,2.60]
Wealth Status				
Poorest	1.50** [1.17,1.91]	2.68** [1.28,5.60]	1.27 [0.93,1.74]	2.17 [0.87,5.45]
Poorer	1.41** [1.10,1.80]	2.21* [1.05,4.66]	1.34 [0.98,1.83]	1.06 [0.56,2.01]
Middle	1.26 [1.00,1.59]	2.06 [0.97,4.38]	1.19 [0.89,1.60]	0.88 [0.50,1.54]
Richer	0.96 [0.79,1.18]	1.69 [0.80,3.56]	0.84 [0.64,1.10]	1.12 [0.76,1.66]
Richest	Reference	Reference	Reference	Reference
Health Insurance				
No Health Insurance	1.26* [1.03,1.53]	1.22 [0.66,2.25]	1.29 [1.00,1.67]	1.34 [0.90,2.00]
Health Insurance	Reference	Reference	Reference	Reference
Mother’s BMI				
Under Weight	0.97 [0.80,1.18]	1.08 [0.69,1.67]	0.9 [0.71,1.15]	0.99 [0.58,1.69]
Normal	Reference	Reference	Reference	Reference
Overweight	0.79*** [0.69,0.89]	0.59*** [0.44,0.79]	0.86 [0.73,1.01]	0.75 [0.55,1.03]
Severe Obesity	0.62* [0.43,0.89]	0.21* [0.05,0.90]	0.54* [0.33,0.88]	0.89 [0.45,1.77]
Birth Type				
Single	Reference	Reference	Reference	Reference
Multiple	1.38* [1.01,1.87]	1.62 [0.92,2.84]	1.09 [0.74,1.60]	2.62 [0.90,7.57]
Child’s Sex				
Male	Reference	Reference	Reference	Reference
Female	0.84*** [0.76,0.93]	0.91 [0.73,1.13]	0.78*** [0.69,0.89]	0.97 [0.74,1.28]
Child’s Age				
6–23 months	Reference	Reference	Reference	Reference
24–47 months	0.37*** [0.33,0.42]	0.52*** [0.40,0.66]	0.35*** [0.31,0.41]	0.30*** [0.22,0.41]
48–59 months	0.27*** [0.23,0.31]	0.36*** [0.27,0.49]	0.25*** [0.21,0.30]	0.19*** [0.12,0.29]
Child’s BMI				
Low	1.39* [1.05,1.85]	2.32** [1.24,4.36]	1.34 [0.95,1.88]	0.62 [0.30,1.28]
Normal	Reference	Reference	Reference	Reference
Overweight	1.21** [1.05,1.40]	0.99 [0.74,1.33]	1.19 [0.99,1.42]	1.87*** [1.29,2.71]
Obese	1.07 [0.84,1.36]	1.52 [0.87,2.67]	0.85 [0.63,1.15]	1.57 [0.81,3.04]
*/cut1*	*0.79 [0.58,1.08]*	*0.81 [0.28,2.31]*	*0.71 [0.46,1.1]*	*0.86 [0.39,1.87]*
*/cut2*	*2.70 [1.97,3.7]*	*2.73 [0.96,7.72]*	*2.42 [1.56,3.74]*	*3.42 [1.57,7.44]*
*/cut3*	*82.9 [57.2120.2]*	*75.0 [25.0,225.3]*	*77.3 [46.9127.3]*	*143.0 [44.4460.6]*
*AIC*	*16,881.3*	*3859.96*	*10,794.61*	*2212.41*
*Log pseudolikelihood*	*− 8411.65*	*− 1902.98*	*− 5370.3*	*− 1079.21*

* *p* < 0.05, ** *p* < 0.01, *** *p* < 0.001

The combined risk of severe, mild or moderate anaemia is significantly higher among children whose mothers reside in the urban [AOR: 1.76, 95%CI: 1.30–2.38] and rural [AOR: 1.39, 95%CI: 1.16–1.66] Zanzibar when compared to children whose mothers resides in urban mainland. Non-access to or non-ownership of health insurance [AOR: 1.26, 95%CI: 1.03–1.53] is associated with a higher risk of anaemia among the children. Maternal overweight [AOR: 0.79, 95%CI: 0.69–0.89] and obesity [AOR: 0.62, 95%CI: 0.43–0.89] compared to a moderate/normal body mass index was significantly associated with a reduced risk of anaemia among children under 5 years.

The risk of anaemia is significantly higher among children living in the poorest [AOR: 1.50, 95%CI: 1.17–1.91], and poorer [AOR: 1.41, 95%CI: 1.10–1.80] households compared to those living in the richest households. Underweight [AOR:1.39, 95%CI: 1.05–1.85] and overweight [AOR:1.21, 95%CI: 1.05–1.40] children are significantly at risk of anaemia compared to children with a normal body mass. Older children aged 24–47 months [AOR:0.37, 95%CI: 0.33–0.42] and those between 48 and 59 months old [AOR:0.27, 95%CI: 0.23–0.31] are significantly less likely to be anaemic compared to children under 24 months old. Female children [AOR:0.84, 95%CI: 0.76–0.93] had a significantly lower risk of anaemia compared to males.

### The role of maternal education in reducing Anaemia risks among children under five years

In order to understand whether having an educated mother can offset the risk of anaemia associated with having a socio-economically disadvantaged mother, we also present in Table 3, the results from our sub-group analysis by level of educational attainment.

We observe no statistically significant difference in the risk of anaemia in almost all the maternal socio-demographic categories including age, employment status, wealth status, health insurance or body mass, particularly among children of women with secondary or higher education. However, maternal residence in urban Zanzibar [AOR:2.28, 95%CI: 1.48–3.54] or rural Zanzibar [AOR:1.84, 95%CI: 1.30–2.60] remains significantly associated with the risk of anaemia among under-five children even with higher levels of maternal education.

Although we found no statistical evidence that marital status was associated with the risk of anaemia in the main model, results from Table 3 shows that children of unmarried mothers [AOR:4.04, 95%CI: 1.98–8.24] with no formal education were about four times more likely to be anaemic compared to children of currently married women with similar levels of education. Similarly, the children of uneducated mothers residing in the poorest [AOR:2.68, 95%CI: 1.28–5.60] or poorer households [AOR: 2.21, 95%CI: 1.05–4.66] were significantly more likely to be anaemic compared to the children of women with similar levels of education but residing in the richest households. Maternal unemployment [AOR:1.31, 95%CI: 1.15–1.49] also remained significantly associated with anaemia among children of women with no formal education.

## Discussion

In this study, we attempted to identify the maternal socio-demographic characteristics associated with the risks of anaemia as well as how access to educational opportunities for mothers may reduce the risk for children under-five in Tanzania. We observed a high level of anaemia among children under-5 years in Tanzania. This confirms the severity of anaemia as a public health challenge that needs immediate actions and measures in Tanzania based on the WHO criteria. This finding is similar to another study in Tanzania [18]. Prior studies have noted high malaria infection, nutritional deficiencies and sickle cell disease to be contributing factors to this high prevalence [3]. We also noted variations in the severity of anemia by place and region of residence. Our finding that anaemia is more common in Zanzibar is supported by a recent report of the 2014 Tanzania National Nutrition Survey. Although deworming pills and iron folic acid (IFA) supplements could help prevent the risk of anemia and are critical for the reduction of child morbidity and mortality the report suggests that children in the mainland (71%) are more likely to be dewormed against Helminthes or intestinal worms compared to Zanzibar resident children (54%) [19]. About 31% of women aged 15–49 years with children under 5 years of age reported not using iron-folic acid supplementation during pregnancy compared to about 37% of women in the Zanzibar [19].

In this study, maternal education emerges as a significant predictor of anaemia. This finding is consistent with those observed in prior studies in Tanzania [20–24]. Maternal education, particularly at the secondary level, has been linked to improved child health outcomes [13]. This protective benefit of maternal education has been shown to be related to an increased knowledge needed for adequate healthcare and nutrition for children hence its possibility for reducing the risk of anaemia.

Maternal employment status is also associated with anaemia among under-five children in Tanzania, and this may be because working mothers are able to afford quality meal supplements, particularly since one of the major causes of anaemia in developing countries is nutrient deficiency. Unemployment is associated with poor socio-economic status. This is likely to reflect nutritional deficiencies and recurrence of infections which more likely increases the risk of anaemia. This result is similar to a study conducted in Mwanza Tanzania that found that unemployment among caretakers was strongly associated with severe anaemia [3].

We observe that maternal age is negatively associated with anaemia. This result corresponds with results from other studies in Cape Verde and rural Indian communities where children whose mothers were younger were significantly more likely to have anaemia [24–26]. Young mothers may have challenges with child care due to limited resources at their disposal which may subsequently result in poor health outcomes [24, 26]. It is also likely that young mothers are at a disadvantage due to other age-related socio-demographic characteristics like education, employment status and marriage [5]. Our finding that a higher number of siblings is associated with increased anaemia is consistent with those observed in prior studies [24, 27]. A high number of children is likely to impact on women’s ability to feed the children appropriately and subsequently trade quality for quantity in order to meet the needs of every member of the family [5, 27]. A higher number of children ever born per woman is also an indication of frequent pregnancy which may also increase the risk of anaemia. [5]. The wealth index has also been identified to be significantly associated with anaemia in young children in studies conducted in rural India and the United States [28, 29]. A common explanation for this observed pattern of relationship has been that malnutrition, deficiencies in other micronutrients, exposure to biofuel smoke and other unexplained characteristics associated with lower socioeconomic status may be a contributing factor [28–30].

In this study, maternal marital status is not significantly associated with the likelihood of anaemia in the general sample. Recent studies of under-five children in sub-Saharan Africa have shown similar findings where maternal marital status was not significantly associated with child health status [31, 32]. However, when the result is stratified by maternal educational attainment, our finding shows that the health disadvantage of having an unmarried mother is stronger for children whose mother has no education. The risk of anaemia for children whose mother has secondary or higher education is not significantly different across almost all other levels of the maternal socio-demographic characteristics. Similar relationships have been found in prior studies. For instance, Smith-Greenaway [13] in her study showed that premarital childbearing in the context of educational advantage does not bear the negative consequences that it does for children whose mothers are educationally disadvantaged. Moreover, increasing evidence from South Africa confirms that even in the absence of marriage, fathers are involved in the well-being of their children [33, 34]. Building upon Smith-Greenaway’s argument, it is possible that more educated unmarried mothers may be better positioned to receive support not only from a family member with greater resources but also from the child’s father [13]. This finding suggests that improved maternal socio-economic conditions are essential for reducing the risk of anaemia among children under 5 years. As a crucial way for reducing the levels of anaemia, our findings coincide with those of previous studies and emphasize the need to invest in women’s education as a way to enhance child well-being in developing countries [18].

It is however worth noting that information on the inherited disorder of haemoglobin structure among the children included in this study such as the case in sickle cell anaemia was not available in the dataset. As a result, it is possible that for some of the children their level of haemoglobin could have been influenced by genetic makeup rather than maternal socio-demographic characteristics.

## Conclusion

The findings from this study underscore the fact that the prevalence of anaemia among under-five children in Tanzania is high especially in the Zanzibar region. Maternal characteristics including older age, higher education, access to health insurance, being employed and high household wealth are protective factors against anaemia among under-five children in Tanzania. We find that access to secondary or higher maternal education reduces the risks of anaemia among children of disadvantaged mothers. The health disadvantage of being born to an unmarried mother is aggravated only among children of women with no education.

Finally, the key recommendation emerging from this study is that programs aimed at reducing anaemia among children under-5 years in Tanzania especially the National Nutrition Strategy by the Ministry of Health and Social Welfare in the country should give special attention to young, males, and malnourished children. Children of unmarried, uneducated and unemployed mothers should also be targeted.

## Abbreviations

BMI: Body Mass Index
Hb: Hemoglobin
HIV: Human immunodeficiency virus
NBS: National Bureau of Statistics
WHO: World Health Organization
